# Trabecular metal technology (TMT) cups in primary total hip arthroplasty: outcomes and survivorship of a large cohort

**DOI:** 10.1007/s00402-025-06063-9

**Published:** 2025-10-24

**Authors:** Alessandro El Motassime, Lorenzo Fulli, Rudy Sangaletti, Luca Andriollo, Francesco Benazzo, Stefano Marco Paolo Rossi

**Affiliations:** 1 Università Cattolica Del Sacro Cuore, UOC Ortopedia e Traumatologia, Agostino Gemelli University Polyclinic, Rome, Italy; 2 Robotic Unit, UOC Ortopedia e Traumatologia, Fondazione Poliambulanza Istituto Ospedaliero, Brescia, Italy; 3 Alma Mater Europaea, Maribor, Slovenia; 4 Istituto Universitario di Studi Superiori di Pavia, Pavia, Italy; 5 Department of Life Science, Health, and Health Professions, Link Campus University, Rome, Italy

**Keywords:** Total hip replacement, Trabecular metal, Primary implants, Fixation

## Abstract

**Introduction:**

Total hip arthroplasty (THA) is a widely performed procedure presenting substantial functional improvement in patients with hip joint pathology. Cementless acetabular components have well renowned popularity for their potential for long-term durability and bone preservation. Among these implants, trabecular metal technology (TMT), particularly using tantalum, has been introduced to enhance biological fixation and implant longevity.

**Methods:**

This retrospective study analyzed 3464 primary THAs performed using tantalum TMT acetabular cups between 2012 and 2022 at a single high-volume arthroplasty center. Patients included were adults undergoing THA for osteoarthritis, hip dysplasia, post-traumatic arthritis, or avascular necrosis, with a minimum follow-up of 24 months. Clinical outcomes were evaluated using the Harris Hip Score (HHS), and implant survivorship was assessed via Kaplan–Meier analysis.

**Results:**

The mean follow-up was 7.03 years (± 2.58). The mean HHS improved significantly from 46.7 (± 7.2) preoperatively to 91.07 (± 7.8) at final follow-up. The implant survivorship free from any reoperation was 97.78%, while survivorship free from aseptic loosening was 99.89%. Radiographically, mean cup inclination was 41.3° (± 5.9), with signs of potential aseptic loosening in only four cases (0.11%). Complications included 46 infections managed with DAIR/DAPRI and 30 implant-related revisions, primarily due to dislocation or mechanical failure.

**Conclusion:**

Tantalum acetabular components demonstrate excellent mid-term clinical and radiographic performance in a broad patient population, suggesting their utility in both high-demand and compromised bone quality cases. Continued follow-up is necessary to confirm long-term implant survivorship.

## Introduction

Total hip arthroplasty (THA) is one of the most commonly performed orthopedic surgeries and in 2007 it was described as “the operation of the 20th century” [1]. It provides significant improvements in the quality of life for patients suffering from degenerative joint diseases, trauma, or congenital conditions [2]. As the global population ages and the prevalence of conditions like osteoarthritis (OA) increases, the demand for THA is expected to rise significantly in the coming decades [3]. 

Uncemented THA has become a key approach in managing degenerative hip conditions, thanks to its long-term durability and potential for preserving bone [4]. A crucial factor in its success is the design and material composition of the acetabular component, which must provide optimal primary stability and encourage osseointegration [5, 6]. 

The primary challenge with cementless acetabular fixation is achieving stable initial fixation, which is crucial for promoting osseointegration and ensuring long-term durability [7–9]. Researchers have developed porous materials looking for biological support fixation to the bone [10]. 

Various coatings and three-dimensional structures are used to enhance both primary fixation capabilities and secondary osseointegration particularly with trabecular structures such as trabecular titanium technology [8, 9, 11].

Biological bone integration of the implant requires intimate contact between the components and bone and immediate mechanical stability during the operation [12]. 

Since the inaugural report regarding the utilization of tantalum (Ta) in the fabrication of tantalum plates for fracture repair surgery in 1940, tantalum has been extensively employed in the field of bone tissue engineering, encompassing tantalum rods, spinal fusion cages, and dental implants. Prolonged clinical evidence reinforces its effectiveness as a surgical material [13–15]. 

In 1997, Tantalum was introduced, promising enhanced durability compared to the less porous conventional titanium acetabular cups [16]. In the last decades porous tantalum-made acetabular cups and augments have been introduced to improve biological fixation in bone defects and allow the normal centre of rotation to be restored [17]. 

The tantalum coating is recognized as a quintessential example of this third generation of cups. These cups, featuring tantalum coatings, demonstrated exceptional outcomes, distinguished by minimal radiolucent lines and a 100% survival rate over a period of 12 years [18]. 

This study primarily aims to evaluate the mid-term survival rates and, secondarily, the clinical and radiographic outcomes of tantalum acetabular cups that are implanted as primary implants in a high-volume hospital.

## Materials and methods

### Demographics

The study included patients treated between January 2011 and July 2023, with all procedures performed at a single high-volume center specialized in primary and revision arthroplasty surgery.

The study included adult patients which underwent the implant of primary THA due to osteoarthritis, hip dysplasia, or femoral head necrosis.

Exclusion criteria included incomplete preoperative, intraoperative or follow-up data, furthermore patients under 18 years of age were excluded, patients which already had undergone hip surgery were as well excluded, and patients with a follow up period of less than 24months. Patients with a history of oncologic disease or an active oncologic condition were excluded from the study. As previously stated, a minimum follow-up period of 24 months was adopted. Patients who died before reaching this time point were excluded from the study, whereas the remaining individuals were considered as survivors until the date of death or until the occurrence of further surgical procedures related to the implant under investigation.

A total of 3464 hip replacements meeting the inclusion criteria were performed using TMT acetabular cups. Among the patients, 1558 (44.98%) were men and 1906 (55.02%) were women. The acetabular component was implanted on the right side in 1844 cases and on the left side in 1621 cases in this retrospective case series, while there have been 3 cases which underwent a bilateral procedure. The mean age of the patients was 69.7 ± 11.2 SD, ranging from 21 to 97 years.

Indications for surgery were: osteoarthritis, in 2459 hips (71%); dysplasia, in 589 hips (17%); post-traumatic arthritis, in 312 hips (9%); avascular necrosis (AVN), in 104 hips (3%). All operations were performed by three experienced surgeons. Demographic data are summarized in Table 1.

All patients underwent radiographic evaluations employing pelvis anteroposterior views and, lateral views of the affected hip. If deemed necessary, a computed tomography (CT) scan was requested to evaluate the status of bone stock. In instances where there was a suspicion of avascular osteonecrosis, magnetic resonance imaging (MRI) was performed. Furthermore, data pertaining to the types of implants utilized were meticulously gathered.

A digital preoperative planning with Sectra^®^ imaging software (SECTRA AB, Linköping, Sweden) was applied in all cases for implant sizing and centre of rotation and offset calculation.

This study was didn’t need Institutional Review Board approval for its nature of retrospective study with a well-established implant. Written consent was acquired for all patients prior to surgery.

**Table 1:** Demographic characteristics

*N*° THA	3464
M/F	1558/1906
Mean age	69.7 (21–97)
Indication for surgery	2459 OA 589 dysplasia 312 post traumatic OA 104 AVN

### Implants

Uncemented Trabecular Metal Modular Acetabular System acetabular cups were utilized (Zimmer Biomet, Warsaw, IN) for THA in all cases. The porous structure has been meticulously engineered to facilitate biological fixation and vascularization, thereby promoting optimal bone integration. Its modulus of elasticity, akin to that of cancellous bone, contributes to a more physiologically normal loading in order to mitigate stress shielding. The shell of this cup is produced utilizing Trabecular Metal Technology (TMT), specifically composed of pure elemental tantalum, and is designed to endure physiological loads. Additionally, it exhibits a high coefficient of friction in comparison to cancellous bone, ensuring stable initial fixation.

Metal (Metasul, Zimmer, Warsaw, IN) on polyethylene (PE) articulations measuring either 28, 32, or 36 mm, as well as ceramic (BIOLOX forte, CeramTec, Plochingen, DE) on polyethylene (PE) articulations measuring 28–32 mm, were implanted. Ceramic-on-PE bearings were utilized for patients exhibiting metal allergies and for younger patients whose body mass index is below 30 kg/m². Associated femoral stems were as follows: 57.1% CLS (Zimmer-Biomet, Wintertur, Switzerland), 36.3% SL-PLUS MIA™ implant (Smith & Nephew-Endoplus, Neuilly, France), 4.9% Wagner SL Revision (Zimmer-Biomet, Wintertur, Switzerland), 1.7% GTS (Zimmer-Biomet, Wintertur, Switzerland), Table 2.

**Table 2:** Implant characteristics

*Acetabular cup measure*, *n (%)*	
60	59 (1.7)
58	161 (4.6)
56	342 (9.9)
54	576 (16.6)
52	685 (19.8)
50	882 (25.5)
48	679 (19.6)
46	80 (2.3)
*Femoral head measure*, *n(%)*	
36	2593 (74.8)
32	681 (19.7)
28	190 (5.5)
*Femoral head material*, *n(%)*	
Metal	1885 (54.4)
Ceramic	1579 (45.6)
*Femoral stem model*, *n (%)*	
CLS	1979 (57.1)
MIA	1259 (36.3)
Wagner	169 (4.9)
GTS	57 (1.7)

### Surgical technique

The surgical procedure was conducted using a posterolateral approach, positioning the patient in a lateral decubitus stance with the pelvis rigidly fixed, in all THAs. An incision measuring between 8 and 12 centimeters was executed. The acetabulum underwent an over-reaming of 1 mm to achieve line-to-line fixation and to compensate for the surface interference with the TMT acetabular cup. The acetabular component was subsequently implanted, with the inclination and anteversion angles customized to match the patient’s specific anatomy or adjusted accordingly in dysplasia cases.

A posterior capsular reconstruction and reattachment of the external rotators were performed in all cases.

Every patient underwent standard venous thromboembolism (VTE) prophylaxis, which consisted of low-molecular-weight heparin given for four weeks after surgery. Perioperatively, cefazolin was administered as a routine prophylactic antibiotic, provided there were no reported allergies. No drains or catheters were utilized in any case. All patients adhered to a standardized postoperative rehabilitation protocol that involved immediate joint mobilization and weight-bearing with crutches on the day of surgery, as tolerated.

### Follow up

A clinical assessment using the Harris Hip Score (HHS) was performed before surgery and at three, six, and twelve months after the procedure, then annually thereafter. At the same time, a radiographic evaluation was conducted to measure the cup inclination angle and to check for any radiolucent lines, osteolysis, or sclerosis, following the three zones defined by DeLee and Charnley [19]. 

The radiographic assessment included imaging views such as the antero-posterior (AP) projection of the pelvis and the lateral projection of the affected hip, adhering to a standardized protocol to ensure consistency. All measurements were taken by a single observer.

Patients were evaluated for complications during and after surgery. A Kaplan–Meier analysis was employed to estimate survivorship, using acetabular revision due to loosening as the primary endpoint.

Deaths that occurred before 24 months have been considered as lost in follow up.

### Statistical analysis

An independent statistician conducted the statistical analysis utilizing SPSS version 18.0. Continuous variables were reported as means and standard deviations, while categorical variables were expressed as frequency distributions and percentages. To ascertain statistically significant differences, comparisons of continuous variables between preoperative and follow-up phases were performed using the t test. The confidence interval was established at 95%, with significance defined as a p-value of less than 0.05. Survival analysis was executed employing the Kaplan–Meier method, whereby revision surgery was regarded as the failure criterion.

## Results

Data of each patient responding to inclusion criteria were stored and analyzed. As a result of the analysis the mean follow up was 7.03 (SD ± 2.58). The average total HHS showed a significant improvement, rising from 46.7 (SD ± 7.2) before surgery to 91.07 (SD ± 7.8) at the final follow-up.

There were no notable differences in clinical outcomes or survival rates between male and female patients. Significant functional recovery and pain relief were observed in every case. All patients were able to return to their normal daily activities without restrictions, showcasing good mobility and joint function.

**Fig. 1:**
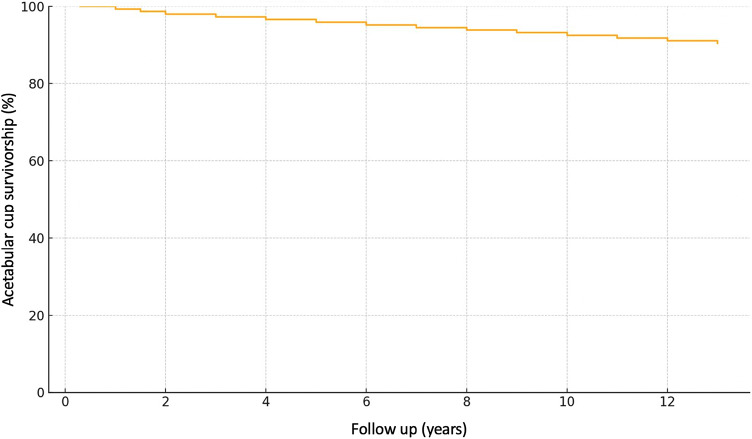
Kaplan–Meier survivorship analysis

76 patients had to undergo further surgery, further analysis showed that of this 76 patients 46 had to undergo further surgery because of infection and underwent DAIR or DAPRI procedures, some of whom underwent multiple surgeries, while the other 30 had to undergo further surgery because of implant problems. This group of 30 patients was further analyzed to study the causes that led to revision surgery which were: dislocation in 20 cases, 4 cases of post traumatic mobilization, 1 case of posterior conflict, 1 case of oxidation of the PE, and 4 cases of mobilization of the implant not connected to any trauma or infection. This resulting in a survivorship with reoperation for any reason of 97.78% and a survivorship specifically related to aseptic loosening of 99.89%. (Fig. 1)

No deaths were recorded within six months following the surgical procedure, and the deaths occurring in subsequent periods were attributed to causes unrelated to the total hip arthroplasty (THA) implantation.

**Fig. 2:**
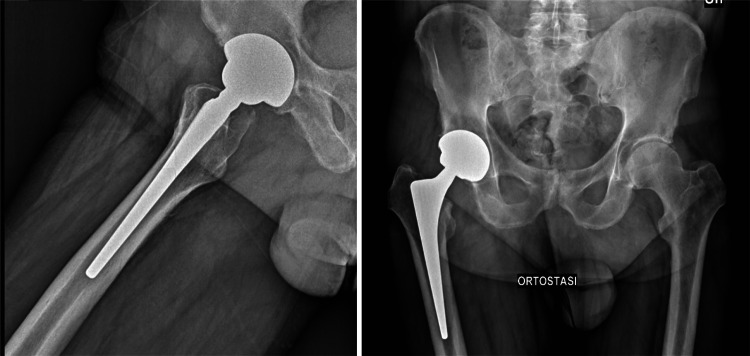
Right TMT acetabular cup associated with a CLS femoral stem

**Fig. 3:**
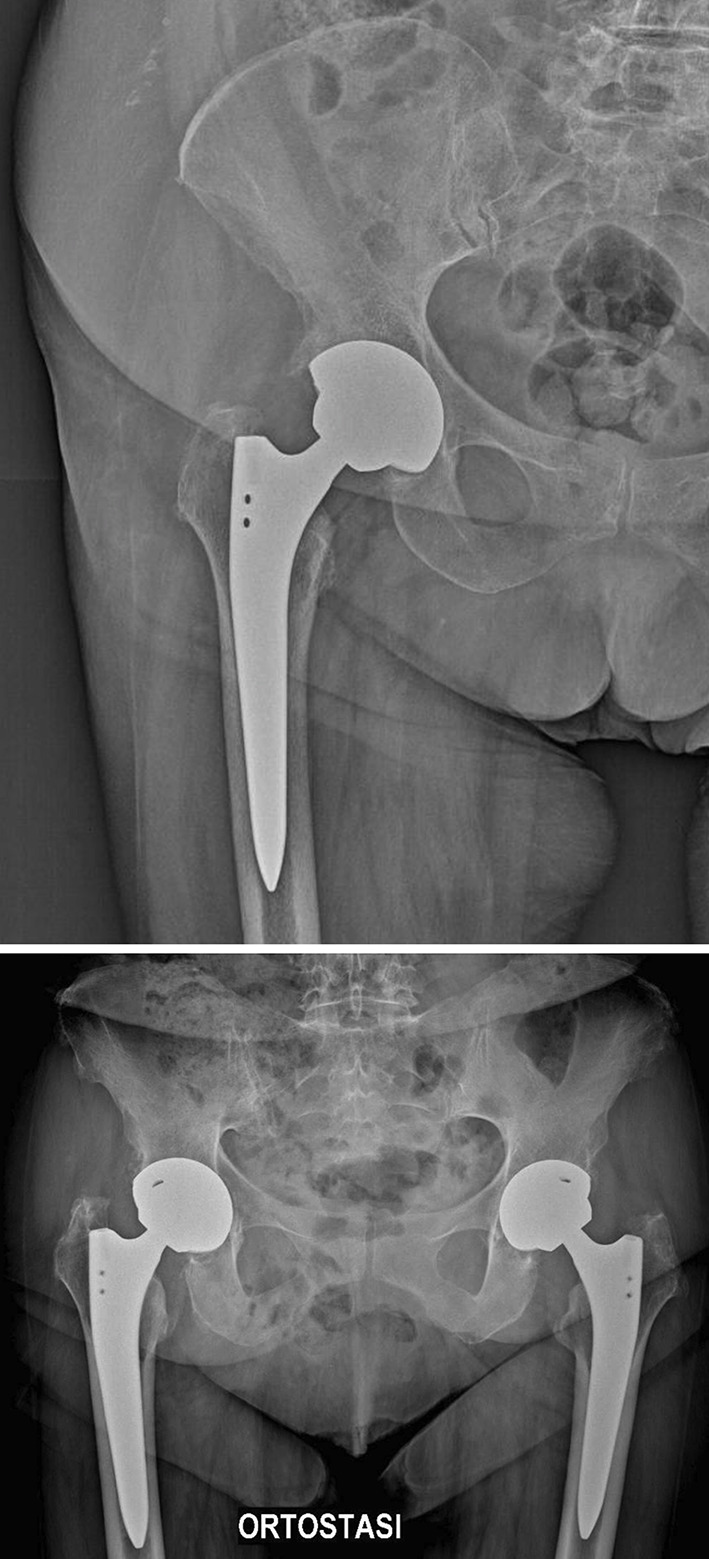
Bilateral TMT acetabular cup implants

Postoperative assessments, performed following the three zones defined by DeLee and Charnley, revealed a mean cup inclination of 41.3° (SD ± 5.9) during the initial radiological evaluation. In four cases (0.11%), X-ray imaging indicated less than 2 mm of radiolucent lines, suggesting possible aseptic loosening. A thorough clinical evaluation followed for all cases. Patients exhibited clinical symptoms, primarily pain and limping, which led to the decision to perform revision surgery. At the final follow-up, all other acetabular components showed radiographic stability, with clear evidence of bone remodeling and osseointegration, and no signs of radiolucent lines, sclerotic areas, or periprosthetic osteolysis observed (Figs. 2 and 3). Results are summarized in Table 3.

**Table 3:** Clinical and radiological outcomes

Follow up	7.03 years (SD ± 2.58)
HHS	46.7 (SD ± 7.2) pre op 91.07(SD ± 7.8) post op
DAIR/DAPRI	46
Implant related complications	20 dislocations 4 post traumatic mobilization 1 posterior conflict 1 oxidation of the PE 4 aseptic loosening
Cup inclination	41.3° (SD ± 5.9)

## Discussion

This study aims to assess the mid-term clinical and radiographic outcomes of primary THA using trabecular metal tantalum hip cups. Our key finding indicates that our patient cohort experienced positive clinical results, alongside a noticeable enhancement in their quality of life. The mean Harris Hip Score improved from 46.7 (SD ± 7.8) to 91.07 (SD ± 7.8) at the final follow-up. Furthermore, this study was primarily aimed at assessing mid term survival of the tantalum TMT acetabular cup, demonstrating an overall survivorship rate of 97.78% and an aseptic loosening related survivorship rate of 99.89% at 7.03 years of mean follow-up (with a maximum monitoring period of 12.9 years). To the best of the authors’ knowledge, this study features the largest patient cohort and the most extensive follow-up data related to trabecular metal tantalum implants. It is particularly important to emphasize that these implants were performed in the context of primary THA, which, in the authors’ view, enhances the significance of this study. This is especially relevant given that the existing literature includes only a limited number of studies focusing on primary implants, most of which involve considerably smaller cohorts or examine implants primarily used in revision settings.

Fundamental to the long-term performance of uncemented acetabular components is to achieve prompt and reliable biological fixation through bone ingrowth [20]. Various surface modifications, such as porous and hydroxyapatite coatings, have been introduced over time to enhance implant integration by increasing the shell’s porosity and improving the bonding interface between bone and prosthesis. More recently, materials that mimic the architecture of cancellous bone, such as trabecular metal made from tantalum, have gained attention due to their favorable mechanical and biological characteristics. Tantalum’s structural properties, including a porosity of approximately 75–80% and an average pore size near 550 μm, closely resemble those of native trabecular bone, offering an optimal scaffold for osseointegration [21]. Its relatively thick coating (2.4–4.2 mm) supports substantial bone infiltration into the implant surface [22]. The specific acetabular component used in our study was designed to promote both immediate mechanical stability and a conducive environment for biological fixation. The combination of a three-dimensional lattice structure and high biocompatibility allows trabecular tantalum to facilitate early bone ingrowth and support bone stock regeneration. For successful long-term outcomes in cementless arthroplasty, both secure initial fixation and the osteoconductive capacity of the implant surface are considered essential.

Two designs of tantalum acetabular cups are available for use in primary total hip arthroplasty: the monoblock and the modular configurations. The monoblock version incorporates a polyethylene liner that is compression-molded directly into the porous tantalum shell, while the modular variant features a separate polyethylene liner, allowing for greater intraoperative flexibility [23, 24]. 

Both configurations of this implant design present distinct advantages and limitations. On one hand, the monoblock acetabular cup eliminates the need for locking mechanisms and dome holes. This design ensures optimal liner-shell conformity, eliminates micromotion at the liner-shell interface, and prevents the ingress of wear particles into the periacetabular region. As a result, it may reduce polyethylene debris generation and thereby lower the risk of periprosthetic osteolysis and aseptic loosening.

On the other hand, the modular design offers greater intraoperative versatility. In cases where optimal bone contact is not achieved, peripheral screw fixation can be employed to enhance initial stability. Furthermore, concerns regarding long-term polyethylene wear, particularly relevant in younger patients, can be addressed with modular components, as the liner can be replaced without revising the entire cup. Additionally, the modular system supports the use of 10°, 20°, or constrained liners, providing surgeons with tailored options for managing complex cases of instability.

Several studies have reported encouraging mid-term outcomes associated with the monoblock design [21, 22, 25]. In the present study, however, the modular version of the tantalum cup was exclusively utilized for all THA procedures due to surgeons’ preferences.

Ensuring reliable initial stability of the implants is essential for osseointegration, which promotes bone ingrowth and reduces the risk of implant failure. Although this study was conducted at a mid-term follow-up, all hips have shown satisfactory fixation and radiographic stability up to this point.

The survival rate related to aseptic loosening of 99.89% observed in our study, conducted over a mean follow-up period of 7.03 years, demonstrates comparability with the survival rates reported in the existing literature. For instance, Macheras et al. in 2017, noted a survival rate of 100% concerning aseptic loosening within a cohort of 140 hips undergoing primary THA performed implanting acetabular cups in trabecular tantalum, with a mean follow-up duration of 18.1 years. Similarly, De Martino et al. in 2016, documented a 100% survival rate for aseptic loosening in a sample of 63 hips implanted with trabecular tantalum acetabular cups in primary THA, with a mean follow-up of 15.6 years. Furthermore, Wegrzyn et al. reported a 100% survival rate regarding aseptic loosening in 45 patients in which the chosen implant for primary THA had a trabecular tantalum acetabular cup, with a mean follow-up of 11.9 years [25–27]. 

From a clinical perspective, a significantly positive to excellent level of pain relief, functional outcomes, and heightened patient satisfaction was observed during the mid-term follow-up. Research undertaken in comparable environments has similarly evidenced remarkable clinical outcomes linked to this technique.

Our functional outcomes, calculated using the HHS, resulted in a mean value of 91.07. When compared to results available in the literature, they appear to be comparable. Indeed, HHS has been widely used to assess patient satisfaction following THA with a tantalum acetabular cup in several studies. Wegrzyn et al. in 2015 reported a mean HHS of 95 ± 4, improving from a preoperative value of 47 ± 9. Similarly, De Martino et al. documented an improvement from preoperative values of 47 points (range 28–57 points) to postoperative values of 94 points (range 65–100 points). Likewise, Macheras et al. reported a mean postoperative HHS of 95.2 ± 6.9, starting from a preoperative value of 44.4 ± 13.4.

It may be worthwhile to further explore and compare the use of this system (and others) in traumatic conditions as well—such as femoral neck fractures—especially in elderly patients, where bone quality is often compromised and materials with high osteointegrative potential are needed as studied by Jannelli et al. [28].

The present study delineates some limitations. First and foremost, all results are reported at the mid-term, necessitating an extended follow-up period to adequately assess the long-term survival of the implants in question. Secondly, the absence of a control or comparison group restricts the robustness of our conclusions; however, we have juxtaposed our findings with other series documented in the literature utilizing the same material. Thirdly, due to the dimension of the cohort no specific demographics or comoribidities that may have influenced the results were taken into consideration. Lastly, a limitation arises from the reliance on standard radiographs for assessing osteolysis, as these modalities typically underestimate the actual incidence and extent of periacetabular osteolysis. The computed tomographic (CT) scan is considered the preferred modality for the detection of these lesions. However, Meneghini et al. conducted a study in 2010 on porous tantalum acetabular cups, which featured a mean follow-up period of 7.7 years, demonstrating the absence of osteolysis in CT scans [6]. These results were corroborated in 2011 by a study carried out by Moen et al., which exhibited a mean follow-up period of 10.3 years [29]. Additionally, the overproduction of artifacts by porous tantalum could obstruct CT scans’ accuracy in evaluating osteolysis and bone ingrowth around the component [30]. 

Moreover, the findings of this study should be considered preliminary, as longer-term follow-up is essential to fully assess implant survivorship over time. With this in mind, we intend to continue monitoring the current cohort of patients who received primary TMT tantalum cups, in order to determine whether the incidence of aseptic loosening increases with extended follow-up.

## Conclusions

Based on our observations, the clinical outcomes derived from a patient population that exhibits a broad spectrum of age indicate that TMT tantalum cups may offer a superior prosthetic solution for both high-demand individuals and elderly patients with lower demands and compromised bone quality. Additional research is warranted to evaluate long-term survivorship.

## Data Availability

Data deposited in a separate data repository and will be available upon reasonable request.

## Abbreviations

AVN: Avascular necrosis
CT: Computed tomography
DAIR: Debridement antibiotic implant retention
DAPRI: Debridement antibiotic pearls retention of implant
HHS: Harris Hip Score
MRI: Magnetic resonance imaging
OA: Osteoasrthritis
PE: Polyethylene
THA: Total hip arthroplasty
TMT: Trabecular metal technology
VTE: Venous thromboembolism
